# Association between early childhood caries and parental educational status among children in Ile-Ife, Nigeria

**DOI:** 10.3389/froh.2025.1581589

**Published:** 2025-10-15

**Authors:** Moréniké Oluwátóyìn Foláyan, Roberto Ariel Abeldaño Zuñiga, Simin Z. Mohebbi, Mohammad R. Khami

**Affiliations:** 1 Research Center for Caries Prevention, Dentistry Research Institute, Tehran University of Medical Sciences, Tehran, Iran; 2 Department of Child Dental Health, Obafemi Awolowo University, Ile-Ife, Nigeria; 3 Postgraduate Department, University of Sierra Sur, Oaxaca, Mexico; 4 Centre for Social Data Science, Faculty of Social Sciences, University of Helsinki, Helsinki, Finland; 5 Community Oral Health Department, School of Dentistry, Tehran University of Medical Sciences, Tehran, Iran

**Keywords:** dental caries, parental education, oral health behaviors, sugar consumption, toothbrushing, fluoridated toothpaste, Sustainable Development Goal 4, Nigeria

## Abstract

**Background:**

Parental educational status is a known risk factor for early childhood caries (ECC). This study explores the association between parental educational status and ECC prevalence among children aged 0–5 years in Ile-Ife, Nigeria, using the Sustainable Development Goal 4 (SDG 4) framework.

**Methods:**

A cross-sectional household survey was conducted between December 2024 and January 2025, involving 1,339 mother–child pairs. Data were collected through structured questionnaires, capturing confounding variables (child's age and sex at birth; infant-feeding profile, such as age at introduction of sugar into the meal; and oral health behavior, such as toothbrushing frequency, use of fluoridated toothpaste, and consumption of refined carbohydrates between meals); independent variable (parental education levels categorized as Qur'anic/primary, secondary, or tertiary); and the dependent variable (ECC determined by the use of the dmft index). A multivariable logistic regression analysis was conducted to determine the associations between the independent and confounding variables and between the independent and dependent variables after adjusting for confounding variables.

**Results:**

The ECC prevalence was 7.3%, with the highest rate observed in children 60–71 months (12.6%). While maternal and paternal education levels showed no direct association with ECC prevalence, they were indirectly linked to ECC through behavioral pathways. Higher maternal education was associated with greater toothbrushing frequency, showing increased odds for both secondary (OR = 3.411, *p* = 0.037) and tertiary education levels (OR = 5.109, *p* = 0.009). However, it was also linked to higher consumption of refined carbohydrates, with secondary (OR = 0.336, *p* = 0.002) and tertiary education (OR = 0.362, *p* = 0.011) showing lower odds of limited intake. Similarly, higher paternal education was positively associated with the use of fluoridated toothpaste—secondary (OR = 2.417, *p* = 0.003) and tertiary (OR = 3.013, *p* = 0.001)—but also corresponded with increased refined carbohydrate consumption (secondary: OR = 0.329, *p* = 0.046).

**Conclusion:**

These findings indicate that while education promotes some protective behaviors, it may also contribute to increased dietary risks mediated by environmental and socioeconomic factors. Context-specific actions are required to align the SDG 4 with efforts to reduce ECC prevalence in vulnerable populations.

## 1 Introduction

Early childhood caries (ECC) is a significant public health issue affecting millions of children worldwide, with profound implications for overall health and well-being (1). Despite advances in dental care and preventive measures, ECC prevalence remains high, particularly among disadvantaged populations (2). Understanding the multifaceted determinants of ECC is crucial for developing effective interventions and policies to mitigate its impact (3).

Prior studies suggested that maternal education is a key factor influencing the risk of ECC—children of mothers with formal education are less likely to consume cariogenic diets, practice poor oral hygiene, or neglect dental services for caries prevention (4–7). In contrast, the association between paternal education and ECC has been less consistently observed, with significant correlations reported less frequently than for maternal education (8).

Parental educational status is a key determinant of children's health outcomes (9). Parental education is a critical factor that influences health behaviors (10, 11) and access to care (12). Behavioral risk factors for ECC, such as hygiene practices (13) and dietary habits (14), are directly influenced by parental knowledge and attitudes, which are often shaped by educational attainment (15–17). The introduction of sugar at an early age (18) and the frequency of sugar consumption (19) are significant risk factors for ECC, highlighting the importance of parental guidance in establishing healthy dietary practices. Demographic factors, including age and sex, further intersect with these determinants to shape the risk of ECC (4).

However, these family-/individual-level factors do not diminish the importance of a supportive environment. Macroeconomic policies, public health initiatives, and social policies are crucial in shaping the conditions in which children grow and develop (20). Policies related to education, healthcare, social protection, labor markets, and housing all play a role in determining access to essential resources and services (21), including parental access to education (22), which can directly impact oral health and the risk for ECC. To be able to propose macroeconomic and social policies and public health initiatives for the prevention of ECC, it is important to identify context-specific relationships between parental educational levels and the prevalence of ECC.

Educated parents are more likely to have stable incomes and better employment opportunities (23), which makes it possible for they to access health information that can reduce the risk of their children developing ECC (24). In addition, with access to modern health information, educated parents are more likely to challenge and modify traditional practices that can also affect the early introduction of children to sugary foods and drinks that increase the risk of ECC (25). In addition, they can afford them access to and use of better healthcare products, including oral hygiene aids (26). On the other hand, parents with lower educational levels may face barriers such as a lack of awareness and financial constraints, leading to inadequate dental care for their children (27).

In Nigeria, the relationship between parental educational levels and the prevalence of ECC is not known. Prior studies had found associations between ECC and maternal oral health knowledge (28) and attitude (29). However, there are no studies linking parental educational status and ECC in Nigeria. It is likely that parental education may influence a unique set of socioeconomic, cultural, and healthcare access factors that may moderate the risk of their children to ECC. Therefore, the current manuscript employed the Sustainable Development Goal 4 (SDG 4) framework to explore the association between parental educational status and ECC. The framework emphasizes inclusive and equitable quality education and lifelong learning opportunities for all. The aim of the study, therefore, is to determine (i) the association between the prevalence of ECC and parental educational status among children in the Ile-Ife Central Local Government Area (LGA) of Nigeria and (ii) the association between parental educational status and the risk factors for ECC among children in the study location. We hypothesize that children of mothers and fathers with higher educational status would have lower odds of experiencing ECC and lower odds of ECC risk behaviors.

## 2 Methods

This study is a subset of a broader cross-sectional investigation aimed at identifying social and physical risk factors associated with ECC in the Ife-Ife Central LGA, a suburban region in Nigeria. Data were collected through a household survey, allowing researchers to reach preschool-aged children, achieve a balanced representation across socioeconomic groups, and obtain a comprehensive reflection of the child population in the study area (30).

### 2.1 Study population

The study population included children 0–5 years, residing with their biological mother or legal guardians, with participation contingent upon obtaining written informed consent from a parent or the guardian. Only children who were present at home during the study period were enrolled, and no exclusion criteria were applied.

### 2.2 Study size

Using the Cochran formula (31), we calculated that the sample size needed for identifying an ECC prevalence of 4.3% (32), using a margin of error of 5% and a confidence level of 95%. The sample size was 64. To recruit 64 children with ECC, the total sample size required for screening was 969. The sample size was increased by 10% to allow for incomplete responses. The planned sample size was 1,066.

### 2.3 Sampling procedure

The study utilized a four-stage cluster sampling design to ensure representativeness and methodological rigor. In the first stage, the Ile-Ife Central LGA was purposively selected due to its established role as a long-term surveillance site for ECC, with population-level data previously collected in 2014 (23) and 2020 (32). In the second stage, 70 enumeration areas (EAs), representing 10% of the 700 EAs demarcated by Nigeria's 2006 National Census, were randomly selected by lottery, reflecting regional norms for household survey representativeness.

In the third stage, households were systematically sampled within each EA by selecting every alternate household along mapped streets. To be eligible, households needed to include at least one child aged 0–5 years living with a biological mother or legal guardian. In the final stage, one eligible mother–child dyad was enrolled per household. Recruitment continued in each EA until the targeted sample size was reached, with the number of dyads per EA allocated proportionally based on population size. Field teams screened and recruited participants until the overall sample target was met. Although the initial sample size calculation required 1,066 participants, the final sample size was 1,411, exceeding the proposed sample size by 32.4%.

### 2.4 Data collection

Data collection took place between December 2024 and January 2025. Data were collected through interviews using a structured questionnaire. Twenty fieldworkers trained for this study administered the questionnaire in the field. They received comprehensive training, which included a 2-day, 2 h online session totaling 4 h, and a 5 h in-person session. The training focused on the research protocol, study questionnaire, ethical considerations, effective communication, and cultural sensitivity during data collection. In addition, the research assistants conducted a pilot study to identify and resolve potential challenges in administering the questionnaire. The questionnaire was translated into Yorùbá, the language of most residents in the local government area.

A team of nine trained and calibrated dentists assessed the dental caries experience of the children. To ensure reliability, clinical examinations were performed on 10 patients, with recordings of caries using the dmft. The Fleiss' kappa inter-examiner reliability score was 0.062 (*p* = 0.349). The dentists underwent further training, including reviewing clinical photographs and repeated practice sessions to diagnose lesions accurately. Their diagnostic competency was refined to align closely with that of the training consultant.

The questionnaires were administered to mothers. The data collected included sociodemographic profiles, children's oral health behaviors, and infant-feeding practices. Intra-oral examinations were conducted to assess the caries status.

### 2.5 Confounding variables

#### 2.5.1 Sociodemographic profile

Information on the age and sex of the children was collected. Age was established as the child's age at their last birthday, and sex was determined as male or female.

#### 2.5.2 Infant-feeding profile

Mothers were asked to estimate the age at which sugar was introduced into their child’s diet by responding to the question: At what age was sugar included in (use name of the child)'s diet? The response options were as follows: at birth (1), within 1 week of birth (2), within 1 month of birth (3), <4 months of birth (4), 4–6 months of birth (5), after 6 months of birth (6), after 9 months of birth (7), after 12 months of birth (8), cannot remember (88), and no response (99). The responses were dichotomized into consumed sugar before 1 year of age (responses 1–7) and consumed sugar 1 year of age and after (response 8). Using the cutoff points, the response to the question was dichotomized into sugar introduction at or after 1 year of age (yes/no).

#### 2.5.3 Oral health behavior

This was assessed through methods previously described (33). Data were collected on toothbrushing frequency, use of fluoridated toothpaste, and consumption of refined carbohydrates between meals for each child. To establish acceptable oral health behavior, the following cutoff points were used based on prior findings in the study population: toothbrushing twice a day or more (34, 35), consistently or almost always using fluoridated toothpaste (35), and consumption of refined carbohydrates in between meals less than three times per day (23). The variables were dichotomized into a “yes” and “no” response. Dental service utilization was not classified as a preventive behavior, as multiple studies had shown that in this population, dental visits were predominantly sought for curative rather than preventive care (36–38). The questionnaire to collect data on oral health behavior of children had been used in the prior survey conducted in the study population (23, 32).

#### 2.5.4 Independent variable

**Parental level of education**: Information was also collected on the *parents'* educational level. The level of education was classified as follows: no formal education, *Quranic* and primary school education, secondary school education, and tertiary education. Parental level of education was categorized in line with the category used in the National Demographic Survey (39).

#### 2.5.5 Dependent variable

**Caries assessment**: ECC was defined as the presence of cavitated or non-cavitated lesions, filled surfaces, or missing primary teeth in children under 72 months of age (40). The dmft index was used to assess ECC prevalence. Each child's dmft score was determined by counting the number of teeth with carious lesions, those extracted due to caries, and those restored with fillings or crowns (41). Carious lesions were, however, defined as the presence of cavitated or non-cavitated lesions and not just cavitated lesions (40).

Parents were asked about any missing teeth not observed during the oral examination, and only extractions due to caries were recorded as missing. The total number of affected teeth provided the dmft score for primary dentition. ECC was categorized as “present” when the dmft score was greater than 0 and “not present” when the dmft score was 0.

Dental caries examination was performed using a plain mouth mirror and a torch as a light source, with the child seated either on the lap of the mother or in a chair. The teeth were examined without prior drying, though gauze was used to remove any gross debris when necessary. The assessment followed a systematic approach, examining each tooth or tooth space sequentially from one to the next.

### 2.6 Data analysis

Descriptive analysis was conducted to determine the prevalence of ECC for the study population. The chi-square test was used to test associations between the prevalence of ECC and (i) the child's age, (ii) the mother's educational level, and (viii) the father's educational level.

In addition, a multivariable logistic regression analysis was conducted to first determine the associations between the independent and confounding variables. A multivariable logistic regression analysis was conducted to determine the association between the independent and dependent variables after adjusting for the confounding variables. These analyses are presented as odds ratios with their respective 95% confidence intervals. All analyses were conducted using SPSS software version 25 at a level of significance of *p* < 0.05.

### 2.7 Ethical consideration

Before commencing the study, ethical approval for the study was obtained from the Institute of Public Health Research Ethics Committee of the Obafemi Awolowo University, Ile-Ife, Nigeria (IPH/OAU/12/2742), and the Tehran University of Medical Sciences, Tehran, Iran (IR.TUMS.DENTISTRY.REC.1402.023). Efforts were made to ensure confidentiality and adherence to ethical principles during fieldwork. All data were collected without the study participants' identifiers (names and residential addresses). There was no compensation for study participation.

## 3 Results

A total of 1,411 mother–child pairs were recruited for the study; of these, complete data were available for 1,339 (94.9%) mother–child pairs and used in the analysis. The age of the mothers/caregivers ranged from 11 years to 62 years, with a mean of 30.18 (SD: 6.0). The age of children ranged from 1 month to 5 years old, with a mean age of 2.6 years (SD: 1.5).

Table 1 shows that maternal education levels varied, with the majority having a secondary education (63.3%). Paternal education followed a similar distribution, with most fathers having secondary education (59.6%). All participants had some level of formal education.

**Table 1:** Early childhood caries profile of children at Ile-Ife central local government area by age, sex, and educational level (*N* = 1,339).

Variables	Categories	*n*	%	ECC				*p*-Value
				Absent		Present		
				*N*	%	*n*	%	
				1,241	92.7	98	7.3	
Age	0–11 months	127	9.5	125	98.4	2	1.6	<0.001
	12–23 months	244	18.2	239	98.0	5	2.0	
	24–35 months	245	18.3	233	95.1	12	4.9	
	36–47 months	248	18.5	225	90.7	23	9.3	
	48–59 months	285	21.3	253	88.8	32	11.2	
	60–71 months	190	14.2	166	87.4	24	12.6	
Sex	Male	649	48.5	600	92.4	49	7.6	0.417
	Female	690	51.5	641	92.9	49	7.1	
Mother's educational level	Quranic or primary	159	11.9	147	92.5	12	7.5	0.125
	Secondary	848	63.3	778	91.7	70	8.3	
	Tertiary	332	24.8	316	95.2	16	4.8	
Father's educational level	Quranic or primary	87	6.5	80	92.0	7	8.0	0.007
	Secondary	798	59.6	726	91.0	72	9.0	
	Tertiary	454	33.9	435	95.8	19	4.2	

Of the 1,339 children, 98 (7.3%) had ECC. The prevalence of ECC varied significantly across age groups, with the lowest occurrence in infants aged 0–11 months (1.6%) and the highest among children aged 60–71 months (12.6%). The differences in the prevalence of ECC by age were statistically significant (*p* < 0.001). The differences in the prevalence of ECC by sex were, however, not statistically significant (*p* < 0.001).

ECC was most prevalent among children whose mothers had a secondary education (8.3%) and least common among those with a tertiary education (4.8%), though the association was not statistically significant (*p* = 0.125). In addition, ECC was most prevalent among children of fathers with a secondary education (9.0%) and least common among those with tertiary education (4.2%), and this association was statistically significant (*p* = 0.007).

Table 2 shows the results of the analysis of associations between parental education levels and key risk factors for ECC. There were no significant associations between parental education levels and the age of sugar introduction.

**Table 2:** Associations between parental educational level and risk factors for early childhood caries.

Categories	Use of fluoridated toothpaste (*n* = 1,212)			Toothbrushing twice a day or more (*n* = 1,270)			Sugar introduction at or after 1 year of age (*n* = 960)			The frequency of refined carbohydrate consumption is less than three times a day. (*n* = 1,278)		
	OR	95% CI	*p*-Value	OR	95% CI	*p*-Value	OR	95% CI	*p*-Value	OR	95% CI	*p*-Value
Mother's educational level												
Quranic or Primary	1.00	–	–	1.00	–	–	1.00	–	–	1.00	–	–
Secondary	1.012	0.637–1.609	0.960	3.411	1.076–10.808	0.037	0.638	0.396–1.027	0.064	0.336	0.170–0.664	0.002
Tertiary	1.495	0.825–2.711	0.185	5.109	1.496–17.440	0.009	0.576	0.319–1.041	0.068	0.362	0.166–0.790	0.011
Father's educational level												
Quranic or Primary	1.00	–	–	1.00	–	–	1.00	–	–	1.00	–	–
Secondary	2.417	1.348–4.332	0.003	1.024	0.268–3.922	0.972	0.779	0.401–1.513	0.460	0.329	0.110–0.979	0.046
Tertiary	3.013	1.550–5.857	0.001	1.572	0.394–6.272	0.522	0.881	0.424–1.830	0.735	0.401	0.129–1.248	0.115

However, mothers with secondary (OR = 3.411, *p* = 0.037) and tertiary (OR = 5.109, *p* = 0.009) education had significantly higher odds of reporting toothbrushing twice a day or more for their children compared with those with Quranic or primary education. In addition, mothers with secondary (OR = 0.336, *p* = 0.002) and tertiary (OR = 0.166, *p* = 0.001) education had significantly lower odds of reporting the consumption of refined carbohydrate in between meals less than three times a day for their children compared with those with Quranic or primary education.

On the other hand, fathers with secondary (OR = 2.417, *p* = 0.003) and tertiary education (OR = 3.013, *p* = 0.001) had significantly higher odds of reporting using fluoridated toothpaste for their children compared with those with only Quranic or primary education. In addition, fathers with secondary education had significantly lower odds of reporting the consumption of refined carbohydrate in between meals less than three times a day for their children compared with those with Quranic or primary education. (OR = 0.110, *p* = 0.046).

Table 3 shows that maternal and paternal education levels were not significantly associated with ECC prevalence, although tertiary education for fathers showed lower odds of ECC prevalence [adjusted odds ratio (AOR): 0.281; 95% CI: 0.061–1.303; *p* = 0.105], while tertiary education for mothers showed higher odds for ECC prevalence (AOR: 1.084; 95% CI: 0.311–3.775; *p* = 0.899).

**Table 3:** Factors associated with the prevalence of early childhood caries (*N* = 878).

Variables	Prevalence of ECC			
	AOR	95% CI lower bound	95% CI upper bound	*p* value
Age				
0–5 years (as continuous variable)	1.515	1.246	1.841	<0.001
Sex				
Females	1.000	–	–	–
Males	1.033	0.623	1.714	0.899
Use of fluoridated toothpaste				
No	1.000	–	–	–
Yes	1.171	0.629	2.183	0.618
Toothbrushing twice a day or more				
No	1.000	–	–	–
Yes	1.746	0.827	3.683	0.144
Age of sugar introduction ≥ 1 year				
No	1.000	–	–	–
Yes	1.330	0.795	2.223	0.277
Frequency of refined carbohydrate consumption is less than three times a day				
No	1.000	–	–	–
Yes	0.431	0.249	0.745	0.003
Mother's educational level				
Quranic or primary	1.000	–	–	–
Secondary	1.070	0.437	2.618	0.882
Tertiary	1.084	0.311	3.775	0.899
Father's educational level				
Quranic or primary	1.000	–	–	–
Secondary	0.885	0.245	3.195	0.885
Tertiary	0.281	0.061	1.303	0.105

## 4 Discussion

The findings from this study suggest that higher parental education is associated with improved oral health practices, while lower education levels correlate with risk factors that increase ECC prevalence. Mothers with secondary and tertiary education were more likely to ensure that their children brush their teeth twice daily, introduce sugar into their diet at or after 1 year of age, and increase the frequency of refined carbohydrate consumption. Similarly, fathers with higher education levels seem more likely to influence their children's use of fluoridated toothpaste and increase refined carbohydrate consumption. Ironically, however, neither the maternal nor paternal educational status was significantly associated with the presence of ECC. These findings highlight that parental education may have an indirect rather than a direct effect on the prevalence of ECC, thereby partially supporting the study's hypothesis.

This study has several strengths. It utilizes a large and representative sample of mother–child pairs, enhancing the reliability of its findings. The incorporation of the Sustainable Development Goal 4 (SDG 4) framework adds a valuable perspective by situating parental education as a social determinant of oral health. The study also employs robust statistical analyses to explore the association between parental education levels and ECC, providing insights into key risk factors such as fluoridated toothpaste use, toothbrushing frequency, and dietary habits. In addition, the study accounts for both maternal and paternal education, allowing for a more comprehensive understanding of how parental education influences oral health behaviors.

However, the study has limitations. The cross-sectional design prevents the establishment of causality, meaning that while associations can be identified, the direction of influence cannot be confirmed. In addition, the self-reported data on oral health behaviors and dietary habits may introduce recall or social desirability biases. Moreover, the absence of a post-training inter-examiner reliability assessment, following an initially moderate Fleiss' kappa score, may raise concerns about the study's internal validity and reproducibility. However, the additional calibration training focused on identified inter-rater gaps to improve examiner alignment and reduce measurement bias, thereby partially mitigating this limitation. Furthermore, while the study highlights significant associations between parental education and oral health behaviors, the direct link between parental education and ECC prevalence was not consistently observed, suggesting that other environmental, genetic, or socioeconomic factors may also play a role. Despite these limitations, the current study provides valuable insights into the role of parental education in shaping children's oral health behaviors.

First, the study findings indicate a significant increase in the prevalence of ECC in the study population over the past 5 years. Previously, the prevalence of ECC, as determined using the International Caries Detection and Assessment System (ICDAS) criteria, was 4.7% (34). The current study reveals a 55.3% increase in ECC prevalence in the last 5 years in the study environment, highlighting a concerning upward trend. Despite this, the rise is still lower than the prevalence of 30% reported in Africa (42) and for other countries in Africa such as 31.7% in Namibia (43), 44.94%–51.72% in South Africa (44), 59.5% in Kenya (45), 17.6% in Uganda (46), and 30.1% in Tanzania (47). This rise warrants further investigation to identify the underlying causes. One potential contributing factor is the absence of a structured national oral health program, despite the existence of a national oral health policy (48). The lack of implementation and enforcement of this policy may have limited the effectiveness of preventive measures and public health interventions aimed at reducing ECC. In addition, other factors such as changes in dietary habits, increased consumption of sugary foods and beverages, limited access to oral health education, and inadequate dental care services could be driving this increase. Further research is needed to explore these factors in greater depth and to inform evidence-based strategies to curb the rising prevalence of ECC. The development and implementation of targeted oral health programs, community education initiatives, and the implementation of the national oral health policies that promote access to preventive and curative dental care for children may help address this growing problem.

Second, a key finding of the current study is the potential pathways through which parental educational status may moderate the ECC experience. Maternal education seems to moderate the risk of ECC in the study population primarily through behavioral modifications. Consistent with prior studies, higher maternal education in the present study was associated with positive oral health behaviors: a significant increase in children's frequency of toothbrushing (49), and a non-significant delayed introduction of sugar into children's diets (50). These findings agree with prior studies that mothers with higher education levels are more likely to adopt and enforce healthier dietary and oral hygiene practices for their children (5, 51–53).

This observation may be linked to the traditional caregiving role of mothers, who are often the primary decision-makers regarding children's diets and oral hygiene routines (54). Their educational attainment likely enhances their awareness of the importance of oral health, influences the type of food available in the house for consumption (55), and equips them with the knowledge to implement preventive measures (56). These findings underscore the importance of maternal education as a possible protective factor against ECC and highlight the need for targeted oral health education programs aimed at empowering mothers, particularly those with lower educational backgrounds. By addressing knowledge gaps and promoting positive behavioral changes, such interventions could help reduce the prevalence of ECC in vulnerable populations. Further research is needed to explore the specific mechanisms by which maternal education influences oral health outcomes and to develop strategies that leverage these pathways for effective ECC prevention.

Third, this study presents an interesting contrast in the influence of paternal education on children's use of fluoridated toothpaste compared with findings in Australia, where paternal education had no impact on dentifrice selection (57). This variation may stem from cultural and societal differences in parenting roles and responsibilities between the two regions. In this study's context, fathers are more actively involved in household purchasing decisions (58). Given that fluoridated toothpaste is typically more expensive than non-fluoridated alternatives in Nigeria (59, 60), higher paternal education may correlate with increased household purchasing power for fluoridated toothpaste rather than heightened awareness of its oral health benefits for children (61). The findings of the current study highlight the potential influence of paternal education as a protective factor against ECC in this population. It also underscores the importance of considering cultural and contextual factors when designing oral health interventions.

Furthermore, higher parental education, both maternal and paternal education, was associated with a higher frequency of consumption of refined carbohydrates in between meals in the current study, in contrast with past studies (62, 63). This suggests that although education enhances parents' knowledge and awareness about the ills associated with sugar consumption, thereby promoting a delay in sugar introduction into the meals of the children, this does not translate to a sustained behavior for the children in later years. There is little clarity about the reason for this. It is important to explore further if there are cultural factors that influence age-related practices of refined carbohydrate consumption in the study population that can explain the observed findings. This paradox suggests that education alone cannot override Nigeria's obesogenic environment, where commercial snacks are culturally desirable and accessible. Time constraints for working parents may further limit dietary supervision.

The study finding, however, suggests that encouraging children's healthy dietary habits may be more effective when interventions target both fathers and mothers (64). While maternal education plays a key role in shaping children's oral health behaviors (65), the influence of educated fathers should not be underestimated. As primary socializers, parents install essential life skills, including dietary and oral hygiene practices, in their children (66). Implementing targeted educational programs that engage both parents—especially in settings where fathers contribute to household decision-making—could strengthen ECC prevention efforts. This is important in light of the association found between the reduced frequency of consumption of refined carbohydrates and lower ECC prevalence, reinforcing the role of diet as a mediating factor for ECC in the study population. Further research is needed to examine how paternal education specifically influences oral health behaviors and to better understand the cultural factors shaping these practices.

Fourth, despite the observed associations between parental educational status and ECC risk behaviors, one of which is increased refined carbohydrate consumption between meals, the direct link between parental education and ECC prevalence was not significant. This is contrary to findings in Saudi Arabia, where Ellakany et al. (67) investigated the association between parental education, socioeconomic status, and dental caries among 3- to 14-year-old children, reporting a high caries prevalence of 70%. This contrast is also seen in studies from Sri Lanka (68), Japan (69), and China (70). This may reflect a complex interplay of rising disposable income enabling the purchase of commercial snacks, cultural perceptions of such snacks as desirable, time constraints limiting supervision, and limited access to healthy alternatives. In Nigeria, more females live under 50% of the median income (71), and women have short maternal leave for childcare (72–74). These factors are associated with a higher risk for ECC (75, 76). In addition, while educated parents demonstrated knowledge by delaying initial sugar introduction in the current study, sustaining low sugar exposure appears challenging in this obesogenic environment (77). This highlights a critical knowledge–behavior gap requiring context-specific interventions addressing accessibility, cultural norms, and practical parenting support, rather than relying solely on education. This finding suggests that additional environmental or structural factors may influence ECC risk beyond parental education alone.

The present study, framed within the SDG 4 framework, highlights education as a key social determinant of ECC prevention. Higher parental education, both maternal and paternal, is associated with healthier oral health behaviors of children. While maternal education remains the dominant influence, paternal education also plays a role, possibly through their involvement in household decision-making. Children of less-educated parents face a greater risk for ECC and may be more severely affected by the rising prevalence of ECC in the study community.

Aligning with SDG 4, this study underscores the need for policies that improve access to quality education and integrate oral health education into public health initiatives. Nigeria must embed oral health within educational and health systems. Approaches may include training teachers as oral health educators, subsidizing fluoridated toothpaste using father-centric voucher programs, and legislating sugar taxes to fund school prevention initiatives. Furthermore, addressing educational inequities and empowering parents with knowledge can create a supportive environment for reducing ECC prevalence. Targeted interventions that engage both parents, recognizing their complementary roles in shaping children's oral health behaviors, are also needed. In addition, sugar reduction policies and community-led campaigns can promote reduced refined sugar consumption. Training community health workers can also expand preventive care in underserved areas (78). Measurable indicators such as the percentage of schools implementing oral health education curricula or reductions in daily sugar exposure can be used to track progress. These steps align with the National Oral Health Policy (2024–2029) (48) and the national strategic health development plan (79), thereby contributing to fast-tracking the attainment of ECC mitigation goals in the policy. These efforts address the complex interplay of education, behavior, and environmental risks highlighted in this study.

In conclusion, the findings of the current study suggest that parental education plays a crucial, although complex, role in shaping the oral health behaviors of children in the study population. While higher parental education levels could influence the frequency of consumption of refined carbohydrates in between meals, higher maternal education seems to increase the likelihood of the child brushing twice a day or more, while higher paternal education seems to increase the likelihood of the child brushing with fluoridated toothpaste. Despite the associations between parental education and caries risk behavior, parental education was not associated with the prevalence of ECC in the study population. This final finding does not preclude the need for targeted public health intervention. Further longitudinal research is needed to establish causality and explore additional contextual factors influencing the association between parental education level and ECC prevalence.

## Data Availability

The original contributions presented in the study are included in the article/Supplementary Material. Further inquiries can be directed to the corresponding author.

## References

[1] Saikia A Aarthi J Muthu MS Patil SS Anthonappa RP Walia T Sustainable development goals and ending ECC as a public health crisis. Front Public Health. (2022) 10:931243. 10.3389/fpubh.2022.931243 36330110 PMC9624450

[2] Anil S Anand PS. Early childhood caries: prevalence, risk factors, and prevention. Front Pediatr. (2017) 5:157. 10.3389/fped.2017.00157 28770188 PMC5514393

[3] Dhull KS Dutta B Pattanaik S Gupta A Md I Wandile B. Decoding early childhood caries: a comprehensive review navigating the impact of evolving dietary trends in preschoolers. Cureus. (2024) 16(4):e58170. 10.7759/cureus.58170 38741840 PMC11090680

[4] Rai NK Tiwari T. Parental factors influencing the development of early childhood caries in developing nations: a systematic review. Front Public Health. (2018) 6:64. 10.3389/fpubh.2018.00064 29616206 PMC5865069

[5] Peltzer K Mongkolchati A. Severe early childhood caries and social determinants in three-year-old children from Northern Thailand: a birth cohort study. BMC Oral Health. (2015) 15:108. 10.1186/s12903-015-0093-8 26370287 PMC4570638

[6] Zhou Y Lin H Lo E Wong M. Risk indicators for early childhood caries in 2-year-old children in southern China. Aust Dent J. (2010) 56:33–9. 10.1111/j.1834-7819.2010.01280.x 21332738

[7] Feldens CA Giugliani ER Vigo A Vítolo MR. Early feeding practices and severe early childhood caries in four-year-old children from southern Brazil: a birth cohort study. Caries Res. (2010) 44:445–52. 10.1159/000319898 20838043

[8] Folayan MO Coelho EMRB Ayouni I Nguweneza A Al-Batayneh OB Daryanavard H Association between early childhood caries and parental education and the link to Sustainable Development Goal 4: a scoping review. BMC Oral Health. (2024) 24(1):517. 10.1186/s12903-024-04291-w 38698356 PMC11064360

[9] Balaj M York HW Sripada K Besnier E Vonen HD Aravkin A Parental education and inequalities in child mortality: a global systematic review and meta-analysis. Lancet. (2021) 398(10300):608–20. 10.1016/S0140-6736(21)00534-1 34119000 PMC8363948

[10] de Buhr E Tannen A. Parental health literacy and health knowledge, behaviours and outcomes in children: a cross-sectional survey. BMC Public Health. (2020) 20:1096. 10.1186/s12889-020-08881-5 32660459 PMC7359277

[11] Sarkar P Rifat MA Bakshi P Talukdar IH Pechtl SML Lindström Battle T How is parental education associated with infant and young child feeding in Bangladesh? A systematic literature review. BMC Public Health. (2023) 23:510. 10.1186/s12889-023-15173-1 36927525 PMC10022043

[12] Prickett KC Augustine JM. Maternal education and investments in children’s health. J Marriage Fam. (2016) 78(1):7–25. 10.1111/jomf.12253 26778853 PMC4712746

[13] Nepaul P Mahomed O. Influence of parents’ oral health knowledge and attitudes on oral health practices of children (5–12 years) in a rural school in KwaZulu-Natal, South Africa. J Int Soc Prev Community Dent. (2020) 10(5):605–12. 10.4103/jispcd.JISPCD_273_20 33282770 PMC7685284

[14] Yadav SP Meghpara M Marwah N Nigam AG Godhani S Chalana S. Association of early childhood caries with feeding, dietary habits, and oral hygiene practices among rural and urban school children of Jaipur. Int J Clin Pediatr Dent. (2022) 15(3):273–9. 10.5005/jp-journals-10005-2396 35991783 PMC9357528

[15] Bornstein MH Cote LR Haynes OM Hahn CS Park Y. Parenting knowledge: experiential and sociodemographic factors in European American mothers of young children. Dev Psychol. (2010) 46(6):1677–93. 10.1037/a0020677 20836597 PMC3412549

[16] Hess CR Teti DM Hussey-Gardner B. Self-efficacy and parenting of high-risk infants: the moderating role of parent knowledge of infant development. J Appl Dev Psychol. (2004) 25(4):423–37. 10.1016/j.appdev.2004.06.002

[17] Huang KY Caughy MOB Genevro JL Miller TL. Maternal knowledge of child development and quality of parenting among white, African-American, and Hispanic mothers. J Appl Dev Psychol. (2005) 26(2):149–70. 10.1016/j.appdev.2004.12.001

[18] Echeverria MS Schuch HS Cenci MS Motta JVDS Bertoldi AD Britto Correa M Early sugar Introduction associated with early childhood caries occurrence. Caries Res. (2023) 57(2):152–8. 10.1159/000529210 36682347

[19] Andy LPA Helmyati S Amalia R. Nutritional factors associated with early childhood caries: a systematic review and meta-analysis. Saudi Dent J. (2024) 36(3):413–9. 10.1016/j.sdentj.2023.12.001 38525179 PMC10960096

[20] Atashbahar O Sari AA Takian A Olyaeemanesh A Mohamadi E Barakati SH. The impact of social determinants of health on early childhood development: a qualitative context analysis in Iran. BMC Public Health. (2022) 22(1):1149. 35676642 10.1186/s12889-022-13571-5 PMC9178833

[21] Osypuk TL Joshi P Geronimo K Acevedo-Garcia D. Do social and economic policies influence health? A review. Curr Epidemiol Rep. (2014) 1(3):149–64. 10.1007/s40471-014-0013-5 25984439 PMC4429302

[22] Schoeni RF House JS Kaplan GA Pollack H. Making Americans Healthier: Social and Economic Policy as Health Policy. New York: Russell Sage (2008).

[23] Davis-Kean PE Tighe LA Waters NE. The role of parent educational attainment in parenting and children’s development. Curr Dir Psychol Sci. (2021) 30(2):186–92.

[24] Levin KA Davies CA Topping GVA Assaf AV Pitts NB. Inequalities in dental caries of 5-year-old children in Scotland, 1993–2003. Eur J Public Health. (2009) 19(3):337–42. 10.1093/eurpub/ckp035 19307245

[25] Scaglioni S De Cosmi V Ciappolino V Parazzini F Brambilla P Agostoni C. Factors influencing children’s eating behaviours. Nutrients. (2018) 10:706. 10.3390/nu10060706 29857549 PMC6024598

[26] Dumitrescu R Sava-Rosianu R Jumanca D Balean O Damian LR Fratila AD The impact of parental education on schoolchildren’s oral health multicenter cross-sectional study in Romania. Int J Environ Res Public Health. (2022) 19(17):11102. 10.3390/ijerph191711102 36078817 PMC9518154

[27] Minervini G Franco R Marrapodi MM Di Blasio M Ronsivalle V Cicciù M. Children’s oral health and parents’ education status: a cross-sectional study. BMC Oral Health. (2023) 23(1):787. 37875845 10.1186/s12903-023-03424-x PMC10594879

[28] Folayan MO Kolawole KA Oziegbe EO Oyedele T Oshomoji OV Chukwumah NM Prevalence and early childhood caries risk indicators in preschool children in suburban Nigeria. BMC Oral Health. (2015) 15:72. 10.1186/s12903-015-0058-y 26123713 PMC4486704

[29] Abiola Adeniyi A Eyitope Ogunbodede O Sonny Jeboda O Morenike Folayan O. Do maternal factors influence the dental health status of Nigerian pre-school children? Int J Paediatr Dent. (2009) 19:448–54. 19732189 10.1111/j.1365-263X.2009.01019.x

[30] Fisher G Pappas G Limb M. Prospects, problems, and prerequisites for national health examination surveys in developing countries. Soc Sci Med. (1996) 42(12):1639–50. 10.1016/0277-9536(95)00319-3 8783426

[31] Cochran WG. Sampling Techniques. 3rd ed. New York: John Wiley & Sons (1977).

[32] Alade M Folayan MO Tantawi E Oginni M Adeniyi AB Finlayson AA Early childhood caries: are maternal psychosocial factors, decision-making ability, and caries status risk indicators for children in a suburban Nigerian population? BMC Oral Health. (2021) 21(1):73. 10.1186/s12903-020-01324-y 33941156 PMC8094474

[33] Folayan MO Kolawole KA Oyedele T Chukwumah NM Onyejaka N Agbaje H Association between knowledge of caries preventive practices, preventive oral health habits of parents and children, and caries experience in children resident in suburban Nigeria. BMC Oral Health. (2014) 14:156. 10.1186/1472-6831-14-156. Erratum in: *BMC Oral Health*. (2015) 15:62. doi: 10.1186/s12903-015-0044-4. 25516332 PMC4279893

[34] Folayan MO Oginni AB El Tantawi M Finlayson TL Adeniyi A. Epidemiological profile of early childhood caries in a suburban population in Nigeria. BMC Oral Health. (2021) 21(1):415. 10.1186/s12903-021-01780-0 34425793 PMC8383461

[35] Folayan MO Kolawole KA Chukwumah NM Oyedele T Agbaje HO Onyejaka N Use of caries prevention tools and associated caries risk in a suburban population of children in Nigeria. Eur Arch Paediatr Dent. (2016) 17(3):187–93. 10.1007/s40368-016-0227-y 27160760

[36] Folayan MEO Oyedele T Ola D. Factors limiting dental service utilization by pupils in Ile-Ife, Nigeria. Niger J Health Sci. (2013) 13:18–23. Available online at: https://www.researchgate.net/publication/302401238_Factors_limiting_dental_service_utilization_by_pupils_in_Ile-Ife_Nigeria (Accessed July 6, 2025).

[37] Adegbembo AO. Household utilization of dental services in Ibadan, Nigeria. Community Dent Oral Epidemiol. (1994) 22(5 Pt 1):338–9. 10.1111/j.1600-0528.1994.tb02064.x 7813189

[38] Osibogun A. Crises and challenges in the Nigerian health sector. J Community Med Prim Health Care. (2004) 16(2):1–7. 10.4314/jcmphc.v16i2.32406

[39] National Population Commission (NPC) [Nigeria] and ICF. Nigeria Demographic and Health Survey 2018. Abuja, Nigeria, and Rockville, Maryland, USA: NPC and ICF (2019).

[40] Drury TF Horowitz AM Ismail AI Maertens MP Rozier RG Selwitz RH. Diagnosing and reporting early childhood caries for research purposes. A report of a workshop sponsored by the National Institute of Dental and Craniofacial Research, the Health Resources and Services Administration, and the Health Care Financing Administration. J Public Health Dent. (1999) 59(3):192–7. 10.1111/j.1752-7325.1999.tb03268.x 10649591

[41] World Health Organisation (WHO). Oral Health Surveys: Basic Methods. 5th ed. Geneva: WHO (2013).

[42] Maklennan A Borg-Bartolo R Wierichs RJ Esteves-Oliveira M Campus G. A systematic review and meta-analysis on early-childhood-caries global data. BMC Oral Health. (2024) 24(1):835. 10.1186/s12903-024-04605-y 10.1111/j.1752-7325.1999.tb03268.x 39049051 PMC11267837

[43] Thopil A. Risk factors associated with early childhood caries: an epidemiological survey in Mariental, Namibia. (Doctoral dissertation). University of the Western Cape (2013).

[44] Kimmie-Dhansay F Barrie R Naidoo S Roberts T. Prevalence of early childhood caries in South Africa: a systematic review. BMC Oral Health. (2022) 22(1):32. 10.1186/s12903-021-01982-6 35135513 PMC10074718

[45] Njoroge NW Kemoli AM Gatheche LW. Prevalence and pattern of early childhood caries among 3–5-year-olds in Kiambaa, Kenya. East Afr Med J. (2010) 87(3):134–7. 10.4314/eamj.v87i3.62199 23057310

[46] Masumo R Bardsen A Mashoto K Åstrøm AN. Prevalence and socio-behavioral influence of early childhood caries, ECC, and feeding habits among 6–36 months old children in Uganda and Tanzania. BMC Oral Health. (2012) 12:24. 10.1186/1472-6831-12-24 22834770 PMC3434064

[47] Rwakatema DS Ng'ang'a PM. Early childhood caries in Moshi, Tanzania. East Afr Med J. (2010) 87(7):304–10. PMID: 23451550 23451550

[48] Federal Ministry of Health. Nigeria National Oral Health Policy: 2024–2029. Abuja: Federal Ministry of Health (2024).

[49] Kuter B Uzel İ. The influence of maternal factors on Children’s oral health: mothers’ age, education level, toothbrushing habit, and socioeconomic Status. J Pediatr Res. (2020) 7(4):331–5.

[50] Wijtzes AI Jansen W Jansen PW Jaddoe VW Hofman A Raat H. Maternal educational level and preschool children’s consumption of high-calorie snacks and sugar-containing beverages: mediation by the family food environment. Prev Med. (2013) 57(5):607–12. 10.1016/j.ypmed.2013.08.014 23988496

[51] Pinto GDS Azevedo MS Goettems ML Correa MB Pinheiro RT Demarco FF. Are maternal factors predictors for early childhood caries? Results from a cohort in southern Brazil. Braz Dent J. (2017) 28(3):391–7. 10.1590/0103-6440201601047 29297562

[52] Dye BA Vargas CM Lee JJ Magder L Tinanoff N. Assessing the relationship between children’s oral health status and that of their mothers. J Am Dent Assoc. (2011) 142(2):173–83. 10.14219/jada.archive.2011.0061 21282684

[53] Abu Hamila NAA. Early childhood caries and certain risk factors in a sample of children 1–3.5 years in Tanta. Dentistry. (2013) 4:180. 10.4172/2161-1122.1000180

[54] AlGhamdi S Abul-Qumsan G. The effect of mother knowledge on the oral health status of their children. IOSR J Dental Med Sci. (2020) 19(4):29–34. 10.9790/0853-1904032934

[55] Folayan MO Oginni AB El Tantawi M Adeniyi A Alade M Finlayson TL. Association between maternal decision-making and mental health and the nutritional status of children under 6 years of age in suburban Nigeria. BMC Public Health. (2023) 23:1159. 10.1186/s12889-023-16055-2 37322502 PMC10268393

[56] Variyam JN Blaylock J Lin B-H Ralston K Smallwood D. Mother’s nutrition knowledge and children’s dietary intakes. Am J Agric Econ. (1999) 81(2):373–84. 1244588/1244588

[57] Buckeridge A King N Anthonappa R. Relationships between parental education, choice of child dentifrice, and their children’s caries experience. Int J Paediatr Dent. (2021) 31(1):115–21. 10.2307/1244588 10.1111/ipd.12716 32815573

[58] Opara BC Uboegbulam GC. Empirical study of family purchase decision for durable goods: the Nigerian experience. In: International Journal of Arts & Science (IJAS), Conference for Business and Economics. Boston, MA: Harvard Medical School (2015). Available online at: https://www.researchgate.net/publication/289768114_EMPIRICAL_STUDY_OF_FAMILY_PURCHASE_DECISION_FOR_DURABLE_GOODS_THE_NIGERIA_EXPERIENCE (Accessed February 15, 2025).

[59] Adegbulugbe IC Adegbulugbe IC. Factors governing the choice of dentifrices by patients attending the Dental Centre, Lagos University Teaching Hospital. Nig Q J Hosp Med. (2007) 17(1):18–21. 10.4314/nqjhm.v17i1.12535 17688167

[60] Umesi D. Availability of non- and low-fluoride paediatric toothpastes in Nigeria: a need for indigenous affordable formulations. Niger Dent J. (2013) 21(1):21–6. 10.61172/ndj.v21i1.66

[61] Alvey J Divaris K Lytle L Vann WF Lee JY. What child oral health–related behaviors can first-time mothers actualize? A pragmatic prospective study. JDR Clin. Transl. Res. (2019) 5:366–75. 10.1177/2380084419892554 PMC7495947 31835968

[62] Fernández-Alvira JM Mouratidou T Bammann K Hebestreit A Barba G Sieri S Parental education and frequency of food consumption in European children: the IDEFICS study. Public Health Nutr. (2013) 16(3):487–98. 10.1017/S136898001200290X 22687743 PMC10271421

[63] Saldiva SR Venancio SI de Santana AC da Silva Castro AL Escuder MM Giugliani ER. The consumption of unhealthy foods by Brazilian children is influenced by their mother’s educational level. Nutr J. (2014) 13:33. 10.1186/1475-2891-13-33 24708610 PMC4011769

[64] Guerrero AD Chu L Franke T Kuo AA. Father involvement in feeding interactions with their young children. Am J Health Behav. (2016) 40(2):221–30. 10.5993/AJHB.40.2.7 26931754 PMC4878394

[65] Dieng S Cisse D Lombrail P Azogui-Lévy S. Mothers’ oral health literacy and children’s oral health status in Pikine, Senegal: a pilot study. PLoS One. (2020) 15(1):e0226876. 10.1371/journal.pone.0226876 10.5993/AJHB.40.2.7 31971936 PMC6977722

[66] Aiuto R Dioguardi M Caruso S Lipani E Re D Gatto R What do mothers (or caregivers) know about their Children’s oral hygiene? An update of the current evidence. Children. (2022) 9(8):1215. 10.3390/children9081215 36010105 PMC9406871

[67] Ellakany P Madi M Fouda SM Ibrahim M AlHumaid J. The effect of parental education and socioeconomic status on dental caries among Saudi children. Int J Environ Res Public Health. (2021) 18(22):11862. 10.3390/ijerph182211862 34831618 PMC8619270

[68] Nanayakkara V Renzaho A Oldenburg B Ekanayake L. Ethnic and socio-economic disparities in oral health outcomes and quality of life among Sri Lankan preschoolers: a cross-sectional study. Int J Equity Health. (2013) 12:89. 10.1186/1475-9276-12-89 24228941 PMC3830507

[69] Kato H Tanaka K Shimizu K Nagata C Furukawa S Arakawa M Parental occupations, educational levels, and income and prevalence of dental caries in 3-year-old Japanese children. Environ Health Prev Med. (2017) 22(1):80. 10.1186/s12199-017-0688-6 29237397 PMC5729505

[70] Li Y Zhang Y Yang R Zhang Q Zou J Kang D. Associations of social and behavioural factors with early childhood caries in Xiamen city in China. Int J Paediatr Dent. (2011) 21(2):103–11. 10.1111/j.1365-263X.2010.01093.x 21121987

[71] UNWomen—Women Counts. Nigeria, Africa (n.d.). Available online at: https://data.unwomen.org/country/nigeria (Accessed July 6, 2025).

[72] Laws of the Federation of Nigeria (1990 Revised edition). The Laws of the Federation of Nigeria. Revised ed. Portsmouth, UK: Law Revision Committee, Grosvenor Press. (1990). p. 7333–407. Volume X, Cap. 198.

[73] Compendium of Nigerian Labour Laws. Nigeria Employers’ Consultative Association, Lagos, Nigeria. Compendium of Nigerian Social and Labour Laws, 2004–01, pp. 127–201. 2nd ed. Lagos, Nigeria: Nigeria Employers’ Consultative Association (1997). p. 197–241. ISBN: 978- 32575-1-X.

[74] Wage Indicator Network. Maternity and work (2021). Available online at: https://mywage.ng/labour-law/maternity-work (Accessed July 6, 2025).

[75] Folayan MO El Tantawi M Vukovic A Schroth RJ Alade M Mohebbi SZ Governance, maternal well-being, and early childhood caries in 3–5-year-old children. BMC Oral Health. (2020) 20(1):166. 10.1186/s12903-020-01149-9 32503512 PMC7275475

[76] Folayan MO El Tantawi M Vukovic A Schroth R Gaffar B Al-Batayneh OB Women’s economic empowerment, participation in decision-making, and exposure to violence as risk indicators for early childhood caries. BMC Oral Health. (2020) 20:54. 10.1186/s12903-020-1045-5 32066424 PMC7026999

[77] Oyeyemi AL Adegoke BO Oyeyemi AY Deforche B De Bourdeaudhuij I Sallis JF. Environmental factors associated with overweight among adults in Nigeria. Int J Behav Nutr Phys Act. (2012) 9:32. 10.1186/1479-5868-9-32 22452904 PMC3331819

[78] Folayan MO Bhayat A Ndembi N Ishola AG El Tantawi M. Essential role of community health workers in promoting oral health in Africa. J Public Health Afr. (2025) 16(1):782. 10.4102/jphia.v16i1.782 40613102 PMC12223844

[79] Federal Government of Nigeria. National Strategic Health Development Plan II (2018–2022). Available online at: https://ngfrepository.org.ng:8443/jspui/bitstream/123456789/3283/1/SECOND%20NATIONAL%20STRATEGIC%20HEALTH%20DEVELOPMENT%20PLAN%202018%20%E2%80%93%202022.pdf (Accessed July 6, 2025).

